# A Carotenoid Extract from a Southern Italian Cultivar of Pumpkin Triggers Nonprotective Autophagy in Malignant Cells

**DOI:** 10.1155/2017/7468538

**Published:** 2017-12-21

**Authors:** Maria Russo, Stefania Moccia, Stefania Bilotto, Carmela Spagnuolo, Miriana Durante, Marcello Salvatore Lenucci, Giovanni Mita, Maria Grazia Volpe, Rita Patrizia Aquino, Gian Luigi Russo

**Affiliations:** ^1^Istituto di Scienze dell'Alimentazione, Consiglio Nazionale delle Ricerche, 83100 Avellino, Italy; ^2^Istituto di Scienze delle Produzioni Alimentari, Consiglio Nazionale delle Ricerche, 73100 Lecce, Italy; ^3^Dipartimento di Scienze e Tecnologie Biologiche ed Ambientali, Università del Salento, 73100 Lecce, Italy; ^4^Dipartimento di Farmacia, Università di Salerno, Fisciano, Italy

## Abstract

Carotenoids, including *β*-carotene, lycopene, and derivatives, such as retinoic acid, have been studied for their significant antiproliferative and differentiating activity on cancer cells in experimental models and in clinics. We are presenting here data on the mechanism of action of a carotenoid-enriched extract obtained from the pumpkin *Cucurbita moschata*, variety “long of Naples,” on two malignant human cell lines, Caco-2 and SAOs, derived from a colon adenocarcinoma and an osteosarcoma, respectively. The carotenoid extract has been obtained from pumpkin pulp and seeds by supercritical CO_2_ extraction and employed to prepare oil-in-water nanoemulsions. The nanoemulsions, applied at a final carotenoid concentration of 200–400 *μ*g/ml, were not cytotoxic, but induced a delay in cell growth of about 40% in both SAOs and Caco-2 cell lines. This effect was associated with the activation of a “nonprotective” form of autophagy and, in SAOs cells, to the induction of cell differentiation via a mechanism that involved AMPK activation. Our data suggest the presence of a pool of bioactive compounds in the carotenoid-enriched extract, acting additively, or synergistically, to delay cell growth in cancer cells.

## 1. Introduction

In the last two decades, the anticancer properties of phytochemicals raised the interest of many scientists, although preclinical and clinical studies often generated contradictory results. Excellent reviews have been published on this topic [1–4]. Among these, a recent meta-analysis of prospective studies suggested a positive correlation between dietary intake of phytochemicals (800–600 mg/die) and reduced risk of cancer, cardiovascular diseases, and all causes of mortality [2]. Carotenoids represent one of the most largely studied class of phytochemicals, since their biological activities have been associated with positive outcomes in terms of human health [5].

Carotenoids are yellow-orange pigments widespread in nature, responsible for the coloration of different plant organs (e.g., roots, fruits, and flowers), but they are also abundantly present in marine invertebrates and microorganisms [6]. Carotenoids include about 600 isoprenoid compounds containing up to 15 conjugated double bonds divided into the two major groups of carotenes and xanthophylls; the former are pure hydrocarbons such as *β*-carotene, while the latter include their oxygenated derivatives, such as zeaxanthin. About 50 carotenoids are present in the human diet, but only 20 have been identified in the human plasma, among these, the most represented are *β*-carotene and *α*-carotene, lycopene, and cryptoxanthin [7, 8]. In human tissues, carotenoids undergo enzymatic transformation leading to apocarotenoids, such as retinoids, which encompass all natural and synthetic derivatives of vitamin A (all-*trans*-retinol) [8].

The capacity of carotenoids to act as free radical scavengers has been indicated for a long time as the molecular mechanism responsible for their protective effects against oxidative stress occurring in several degenerative diseases [6, 9]. In fact, their chemical structure, characterized by a long system of conjugated double bonds with *π*-electrons delocalized over the length of the polyene chain, results effectively in scavenging reactive oxygen species (ROS) [10]. However, while the *in vitro* activity of carotenoids as ROS quenchers is well-known, their antioxidant capability *in vivo* is still controversial and debated. In particular, the potential prooxidant properties of *β*-carotene have been widely discussed and related to the alarming results of important chemopreventive studies [11]. In fact, in the *β*-Carotene and Retinol Efficacy Trial (CARET) and the ATBC (*α*-tocopherol *β*-carotene) studies, the daily administration of *β*-carotene (30 mg *β*-carotene and 25,000 IU retinyl palmitate in the CARET; 20 mg *β*-carotene plus 50 mg *α*-tocopherol in the ABTC) was associated with an increased risk of lung and prostate cancer development [12, 13]. The results of these studies are examples of the unexpected outcomes of clinical studies based on the administration of phytochemicals in cancer prevention [4]. It has been suggested that dietary dosage of carotenoids may promote health, but supplementation with high doses may be associated with adverse effects in smokers or subjects exposed to environmental pollutants [14]. However, the promising results of a randomized controlled clinical trials emphasize the therapeutic potential of lycopene to lower PSA (prostate-specific antigen) serum levels in prostate cancer [15]. Similarly, in a more recent trial, it has been reported that high concentrations of plasma *β*-carotene and *α*-carotene were associated with lower breast cancer risk in oestrogen-receptor negative tumours [16].

The conflicting results deriving from clinical studies highlight the need to reconsider both preclinical and clinical approaches in the study of the potential beneficial effects of carotenoids and to explore new and unknown mechanisms of actions. Several research papers associate the anticancer effects of specific carotenoids (e.g., *β*-carotene, lycopene, and the retinol metabolite all-*trans* retinoic acid [ATRA]) and not-retinoid precursors of carotenoids (e.g., astaxanthin and fucoxanthin), with a variety of molecular mechanisms: (1) ability to modulate hormone and growth factor signalling; (2) regulation of cell cycle machinery; (3) cell differentiation; (4) apoptosis (type I cell death); and (5) autophagy (type II cell death) [14, 17–21]. Autophagy is a complex process which can modulate cell death or cell survival, depending on the specific physiological conditions; therefore, autophagy has been implicated in the physiopathogenesis of cancer and other degenerative conditions [22, 23]. Cells activate autophagic pathways under stress conditions inducing distinct morphological changes, such as the formation of double-membraned vacuoles, which incorporate parts of the cytoplasm subsequently digested by lysosomal hydrolases. This event is important to maintain cellular integrity through the regeneration of metabolic precursors and the elimination of damaged organelles [22, 24]. Autophagy is essential for tissue homeostasis and embryonal development of multicellular organisms; it is governed by about 30 genes (autophagy-related gene, ATG), first discovered in yeast and, subsequently, in higher vertebrates [25]. The biochemical pathway involved in membrane formation requires two ubiquitin-like conjugation systems: ATG5-ATG12 and LC3-ATG8 (microtubule-associated protein light chain 3). The LC3 system is present in two forms: the inactive, free cytosolic form (LC3-I) and the active form conjugated to phosphatidylethanolamine (LC3-II). The function of ATG5-ATG12 and LC3 system is the complexation to the autophagosome membrane during the extension phase; as a result, the autophagosomes contribute to the downstream events: formation of mature vesicles, their fusion to lysosomes, and, finally, degradation of the cargo [26]. New and more complex roles have been identified for autophagy in cancer cells, where this process can exert opposite effects depending on the cellular context and tumour progression. In particular, four functionally different forms of autophagy can be induced by drug treatment, defined as cytoprotective, nonprotective, cytotoxic, and cytostatic autophagy [27, 28]. Cytoprotective autophagy results in enhancing cancer cell survival since it confers resistance to chemotherapy and increases apoptosis when blocked. However, chemotherapy can also promote a “nonprotective” form of autophagy, which may contrast uncontrolled cell growth and can be associated with cell cycle arrest (cytostatic autophagy) and/or the activation of cellular differentiation. It is important to underline for the comprehension of the present work that inhibition of nonprotective autophagy does not influence drug sensitivity [28]. The role of nonprotective autophagy in cellular differentiation has also been demonstrated in colon adenocarcinoma cell lines (Caco-2 and HT-29), where the heterotrimeric G_i3_ protein regulates autophagy and cell state of differentiation [29]. In addition, inhibition of autophagy suppresses mesenchymal stem cell differentiation to osteoblasts [30–33]. More recently, it has been shown that the activation of differentiation in acute promyelocytic leukemia (APL) blasts and osteosarcoma cell lines by ATRA triggers the autophagic process [30, 34, 35].

In recent years, very few papers have been published on the role of selected carotenoids, such as astaxanthin and fucoxanthin, in the regulation of autophagy in precancerous and cancer cells [17, 18, 21]. The present study investigates the capacity of a supercritical CO_2_ (SC-CO_2_) extract enriched in carotenoids obtained from *Cucurbita moschata* sp. to regulate cell growth in human malignant cells. We tested oil-in-water (o/w) nanoemulsions prepared from the carotenoid-containing extract in two human cancer cell lines: SAOs and Caco-2, derived from a human osteosarcoma and colon adenocarcinoma, respectively. We concluded that the carotenoid-enriched extract, administered to cancer cell lines by nanoemulsions, triggers a “nonprotective” form of autophagy, which, in turns, is associated with a delay in cell growth, and induction of differentiation via a mechanism, which involves AMPK activation.

## 2. Materials and Methods

### 2.1. Sample Origin and Preparation

In the present study, carotenoid-enriched extract was prepared from a typical product of Campania Region (Italy), the pumpkin variety “long Neapolitan pumpkin,” also known as pumpkin “full” of Naples. Long Neapolitan pumpkins were peeled, and flesh of fully ripe fruits was chopped into small pieces and dehydrated, at 60°C, by a SalvisLab IC40 vacuum-drying oven (Bio Instruments S.r.l., Firenze, Italy). Simultaneously, seeds were recovered and dehydrated in the vacuum-drying oven. Dried pumpkin flesh and seeds were ground in a laboratory ultra centrifugal mill (ZM200, Retsch GmbH, Haan, Germany) through 70 mesh (210 lm) or 35 mesh (500 lm) sieves, respectively. After grinding, the oven-dried flesh matrix plus milled seeds (1 : 1, *w*/*w*) were extracted by SC-CO_2_ and the obtained oil was characterized for the main lipophilic molecules (carotenoids, tocochromanols, and fatty acids) as reported [36–38]. Shortly, the total amount of carotenoids was of 49.2 mg/100 g, with *α*-carotene and *β*-carotene representing 38.5 and 46.7% of the total, respectively.

### 2.2. Preparation and Analysis of Nanoemulsions

Oil-in-water (o/w) nanoemulsions containing carotenoid-enriched extract (CEN: carotenoid extract nanoemulsion) were prepared using a high-energy emulsification-evaporation technique [39]. Briefly, the carotenoid-enriched extract was mixed in a 1 : 3 ratio (*v*/*v*) with tetrahydrofuran (THF) containing 0.0025% (*v*/*w*) butylated hydroxytoluene (BHT) and added to an aqueous solution containing 0.3% Tween 80. Organic/aqueous phase volume ratio was 1 : 9. The emulsions were homogenized using an Ultra-Turrax homogenizer (T8, Ika-Werke, Germany) performing three cycles at 10000 ×g for 2 min. To reduce particle size, samples were sonicated (Sonicator, ultrasonic processor XL, Misonix), according to Kentish et al. [40]. The solvent was removed from nanoemulsions under nitrogen vapours, and samples were sterilized throughout a 0.2 *μ*m membrane. Control nanoemulsions (NE) were prepared replacing carotenoid extract with the same quantity of THF/BHT 0.0025% *v*/*w* in the aqueous solution. Particle size (expressed as “derived diameter”) distributions were measured by LALLS (low angle laser light scattering technique), and the concentration of carotenoids incorporated into nanoemulsions was determined by extracting 0.5 ml of nanoemulsions with hexane and absolute ethanol (2 : 1 *v*/*v*) as reported [41].

### 2.3. Cell Culture and Cell Proliferation

Two cell lines, Caco-2 and SAOs, derived from human colorectal adenocarcinoma [42] and osteosarcoma [43, 44], respectively, were employed. For the proliferation assays, cells were maintained at low confluence in polystyrene Petri plates (Corning, Milan, Italy) treated to ensure an optimal adhesion. The culture medium used was Dulbecco's modified Eagle medium (DMEM) enriched to 10% foetal bovine serum (FBS) (Life Technologies, Milan, Italy), 1% L-glutamine, 1% not-essential amino acids, and 1% penicillin/streptomycin solution (Life Technologies). Cells were treated as indicated and incubated at a temperature of 37°C in a humidified atmosphere containing 5% CO_2_. Caco-2 and SAOs cells were incubated at different times (96–168 h and 48–168 h, resp.) with CEN corresponding to a concentration of 400 and 200 *μ*g/ml (*w*/*v*) carotenoid-enriched extract, respectively. At the end of incubation, cell suspension was stained using Trypan blue solution (0.04% *v*/*v*), diluted in the culture medium (1 : 1). The cell count was performed using EVE Automatic cell counter (*Eve*™, NanoEnTek, Seoul, South Korea) and expressed as cell number/ml. CyQuant viability assay was performed to quantify the number of living cells using a nuclear dye that selectively binds to nucleic acids, emitting fluorescence. CyQuant assay was employed to the following treatments: (1) Caco-2 and SAOs cells, treated with CEN at the times and concentration indicated above; (2) SAOs cells added with 0.1 mM AICAR (5-aminoimidazole-4-carboxamide 1-*β*-*D*-ribofuranoside, acadesine, N^1^-(*β*-*D*-ribofuranosyl)-5-aminoimidazole-4-carboxamide; Sigma-Aldrich, Milan, Italy), an AMPK (5′ adenosine monophosphate-activated protein kinase) activator [45]; and (3) SAOs and Caco-2 cells treated with 20 *μ*M chloroquine during the last 24 h of incubation, or pretreated with 50 nM bafilomycin A1 (Sigma-Aldrich) to assess the activation of a nonprotective autophagy, before treatment with CEN at concentration indicated above for 96 h. Briefly, CyQuant mixture, containing the nuclear dye (CyQuant nuclear stain) and the suppressor of basal fluorescence (background suppressor), was added to the culture medium and incubated for 1 h at 37°C. Fluorescence was measured at the excitation wavelength of 485 nm and 530 nm emission, and the results were expressed as percentage of fluorescence of the untreated control using a microplate reader (Synergy HT BioTek, Milan, Italy).

### 2.4. Colony Forming Assay

Colony formation assay was performed as previously described [46]. Briefly, SAOs-2 cells were seeded at low density (1000 cells/well) into a 12-well plate, untreated or treated with CEN 200 *μ*g/ml (*w*/*v*), and cultured at 37°C until the large number of clones presented more than 50 cells (12 days). Cells were then washed in phosphate-buffered saline (PBS), fixed using 10% formaldehyde in PBS, and stained with crystal violet (Sigma-Aldrich). Results were expressed as percentage of absorbance at 595 nm after solubilization with acetic acid with respect to untreated controls using a microplate reader (Synergy HT BioTek). Images of representative colonies were captured in bright field using an inverted microscopy (Zeiss Axiovert 200, Milan, Italy).

### 2.5. Measurement of Autophagy and Autophagic Flux

Autophagy was monitored using the Cyto-ID Autophagy Detection Kit (ENZO Life Science, Milan, Italy) as described [24, 47, 48]. Caco-2 and SAOs cells were incubated for 168 h with CEN (400 and 200 *μ*g/ml (*w*/*v*), resp.) and with 20 *μ*M chloroquine or 2 *μ*M ATRA as positive controls. In particular, we estimated the impaired autophagy flux in SAOs cells by monitoring the accumulation of autophagic compartments induced by chloroquine, a lysosome inhibitor that impedes the fusion of autophagosomes and lysosomes and/or the activity of autolysosomes, adding it the last 4 h of incubation together with different treatments as described [49, 50]. After incubation, cells were washed and incubated with the autophagy detection marker (Cyto-ID) and the nuclear dye (Hoechst 33342). Subsequently, cells were rinsed with assay buffer and photographed using a fluorescence microscope (Zeiss Axiovert 200) and a confocal microscopy (Leica SP8; Leica Microsystems, Milan, Italy). Finally, autophagosomes were quantified by normalizing green (Cyto-ID) and blue (Hoechst) fluorescences using a microplate fluorescence reader (Synergy HT BioTek).

### 2.6. Knockdown of Beclin-1 in SAOs Cell Line

SAOs cells were transfected with small interfering RNA (siRNA) targeting specifically at human Beclin-1 gene (BECN1) (SignalSilence® Beclin-1 siRNA I from Cell Signaling Technology, Milan, Italy) or a nontarget siRNA-FITC (SignalSilence control siRNA fluorescein conjugate from Cell Signaling Technology). The oligonucleotide concentrations (20–40 nM) and time of transfection (72 h) were carefully selected to obtain an efficient protein knockdown with negligible toxicity [51, 52]. si-BECN1 or its negative control (si-FITC) was transfected into SAOs cells using Interferin® (Polyplus-transfection® Strasbourg, France) following the manufacturer instructions. To assess protein knockdown and autophagy functional effects, BECN1 and LC3-I/II expressions were detected by immunoblotting on SAOs cellular lysates prepared as described above. Cells without transfection were used as normal control and compared to si-FITC and si-BECN1 samples untreated or treated with NE or CEN (200 *μ*g/ml *w*/*v*) for 72 h. CyQuant assay was employed to measure cell viability at the end of the experiment.

### 2.7. Immunoblotting

Caco-2 and SAOs cells were incubated with CEN at the same concentrations reported above, in the presence of 2 *μ*M ATRA, as positive control. At the end of incubation, cells were lysed using a lysis buffer containing protease and phosphatase inhibitors, as reported [53]. After the measurement of protein concentration [54], the total protein lysates were loaded on a 4%–12% precast gel (Novex Bis-Tris precast gel 4%–12%; Life Technologies) using MES (2-(Nmorpholino) ethanesulfonic acid) or MOPS [(3-(N-morpholino) propanesulfonic acid)], (50 mM MOPS, 50 mM Tris, 1% SDS, 1 mM EDTA; pH 7) buffer. The immunoblots were performed following standard procedures, using as primary antibodies: anti-LC3, anti-BECN1, anti-pAMPK^Thr172^, anti p27^KIP1^ (Cell Signalling Technology), and anti-*α*-tubulin (Sigma-Aldrich) antibodies. PVDF membranes were finally incubated with horseradish peroxidase-linked secondary antibody raised against mouse or rabbit and immunoblots developed using the ECL Plus Western blotting detection system kit (GE Healthcare, Milan, Italy). Band intensities were quantified measuring optical density on a Gel Doc 2000 Apparatus (Bio-Rad Laboratories, Milan, Italy) and multianalyst software (Bio-Rad Laboratories).

### 2.8. Alkaline Phosphatase Activity

Alkaline phosphatase activity (ALP) was measured in total cell extracts using as substrate p-nitrophenyl phosphate (p-NPP; Sigma-Aldrich) [55]. Briefly, SAOs cell extract (5 *μ*g) was incubated at 37°C for 10 min in a reaction buffer containing 10 mM p-NPP and 5 mM MgCl_2_ in 100 mM Tris/HCl pH 9.5. The reaction was terminated by adding 1 N NaOH. The extent of hydrolysis was measured spectrophotometrically at 405 nm, and results were expressed as O.D. values/min/*μ*g protein.

### 2.9. Intracellular ATP Levels

To measure intracellular ATP levels, we used the ViaLight plus kit (Lonza; Euroclone, Milan, Italy) based on the bioluminescence measurement of ATP in metabolically active cells. According to the manufacturer instructions, SAOs cells (2500/well) were seeded in 96-well microtitre tissue culture plate (Costar). At the end of incubation with CEN (200 *μ*g/ml, *w*/*v*) and control nanonemulsions, in the presence of 2 *μ*M ATRA, medium was removed and cells were treated with 50 *μ*l of lysis buffer for 10 min at room temperature. Subsequently, the kit reaction buffer was added and samples incubated for 2 min at room temperature. Finally, the plate was placed in a microplate luminometer (Synergy HT) and results expressed as nmol of ATP after extrapolation of luminescence values using a linear calibration curve made using standard ATP [56].

### 2.10. Statistical Analysis

Data are presented as mean values ± standard deviation (SD), and the significance between the treated group (indicated as CEN) and the control groups (vehicle or untreated) was measured using the Student's test of at least five determinations.

## 3. Results and Discussion

### 3.1. Antiproliferative Effect of CEN on Caco-2 and SAOs Cell Lines

The two cell lines employed in the present study have been selected after a careful screening of different cell lines (data not shown) for the following reasons: (1) high resistance to different cell death stimuli (e.g., *γ*-radiations and treatment with death ligands); (2) ability to differentiate *in vitro* when treated with bioactive compounds; and (3) low toxicity of the vehicle (NE) employed to prepare the carotenoid-enriched nanoemulsions [34, 57].

**Figure 1:**
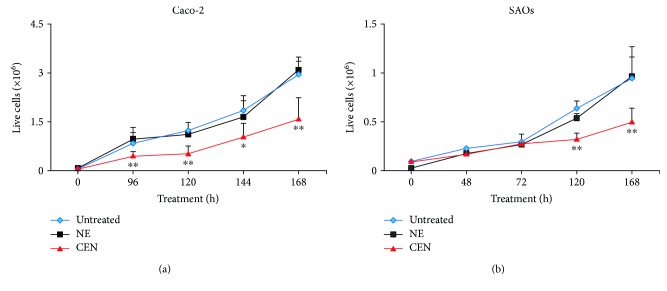
Effects on cell growth of CEN on Caco-2 (a) and SAOs (b) cell lines. Cells were treated with different doses of NE and CEN (400 and 200 *μ*g/ml, *w*/*v*, resp.) and counted at the indicated times using Trypan blue method, as described in Materials and Methods. Line graphs represent the mean of three experiments (±SD). Symbols indicate significance: ^∗^*p* < 0.05 and ^∗∗^*p* < 0.01 with respect to untreated and NE.

Caco-2 and SAOs cells were incubated with CEN, corresponding to 400 and 200 *μ*g/ml of carotenoid-enriched extract (*w*/*v*), respectively, at different times. The effect on cell growth is reported in Figure 1 which shows that the treatment of Caco-2 with CEN significantly delayed cell proliferation of about 50%, starting from 96 h of incubation, compared to controls (untreated and NE; Figure 1(a)). The same effect was observed in SAOs cells where the decreased rate of cell growth (40%) was significant starting from 120 h (Figure 1(b)). The two different CEN concentrations applied, for example, 400 and 200 *μ*g/ml (*w*/*v*) for Caco-2 and SAOs, respectively, were established in preliminary dose-response experiments and selected to minimize the toxicity of the vehicle (NE; data not shown).

The different dose- and time-response observed in the two cell lines employed can be *bona fide* attributed to the differences existing in Caco-2 versus SAOs cells in terms of (1) duplication time, slower in the latter; (2) different uptake and metabolism of CEN; and (3) differences in signalling pathways sensitive to CEN components. Finally, it is worthwhile to note that the effect on the rate of cell growth, following CEN treatment, cannot be attributed solely to *β*-carotene, the major carotenoid in CEN [36, 38], since when *β*-carotene was assayed at the same concentration present in CEN, we did not measure any significant effect on cell growth (data not shown).

**Figure 2:**
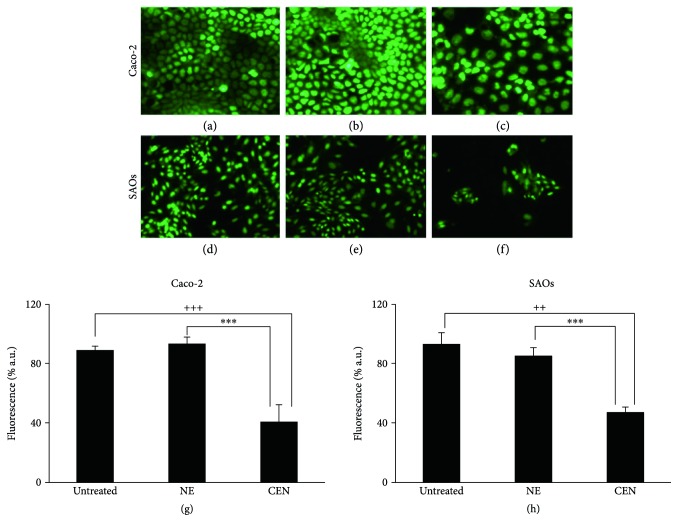
CEN delays cell proliferation in Caco-2 and SAOs cells. Micrographs of cell nuclei stained with CyQuant fluorescent dye of Caco-2 (a, b, c) and SAOs (d, e, f) after 168 h of treatment with NE and CEN (400 and 200 *μ*g/ml, *w*/*v*) (microscope Axiovert 200 Zeiss; FITC fluorescence filter, 200x magnification). Representative images of untreated Caco-2 and SAOs cells: untreated (a, d), incubated with NE (b, e) or CEN (c, f), respectively. CyQuant assay for nuclei quantification is shown in (g) for of Caco-2 and (h) for SAOs cells. CyQuant assay was performed after 168 h of treatment, as described in Materials and Methods. Bar graphs represent the mean ± SD; symbols indicate significance: ^∗∗∗^*p* < 0.001 with respect to NE; ^+++^*p* < 0.001; and ^++^*p* < 0.01 with respect to untreated cells.

The delay in cell growth was also confirmed by CyQuant viability assay. The representative micrographs of the cell nuclei, performed by fluorescence microscopy, show the reduced number of viable cells in Caco-2 (Figure 2(c)) and SAOs (Figure 2(f)) after treatment with CEN at 168 h, compared to NE (Figures 2(b) and 2(e)) and untreated cells (Figures 2(a) and 2(d)). Figures 2(g) and 2(h) show the quantification of data represented in Figures 2(a)–2(f), measured as percentage of emitted fluorescence. CEN significantly slowed down the cell growth of 57% in Caco-2 and of 45% in SAOs cell lines.

**Figure 3:**
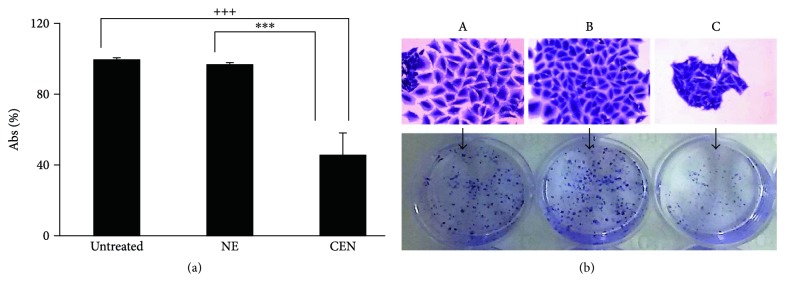
CEN slowdowns cell proliferation. SAOs cells were treated with NE and CEN (200 *μ*g/ml, *w*/*v*) for 12 days. Colony forming assay was performed at the end of incubation as described in Materials and Methods and quantitated in the graph shown in (a). Bar graphs represent the mean ± SD; symbols indicate significance with ^∗∗∗^*p* < 0.001 with respect to NE and ^+++^*p* < 0.001 with respect to untreated cells. (b) Representative photographs are reported of single colony (top) and total colonies (bottom) of untreated (A) cells and those treated with NE (B) and CEN (C) (200 *μ*g/ml, *w*/*v*).

It is worthwhile to note that we did not detect any sign of cytotoxicity (e.g., apoptosis and necrosis) following treatment of both cell lines with CEN (data not shown). To further confirm the capacity of CEN to delay cell proliferation without affecting cell viability and inducing apoptosis, we performed a colony formation assay, which gave an indication of CEN effect of CEN after prolonged treatment. As reported in Figure 3, the number of SAOs colonies formed after treatment with CEN was reduced of about 60%.

### 3.2. Activation of Autophagy by the Carotenoid-Enriched Extract

We hypothesized that the reduced rate of cell proliferation induced by CEN, in the absence of any “signature” of cell death, could be related to the activation of an autophagic process. This hypothesis was also comforted by the microscopically observation of CEN-treated cells, which evidenced the presence of intracellular vacuoles. This hypothesis was investigated using multiple assays to detect autophagy and exclude false-positive results.

**Figure 4:**
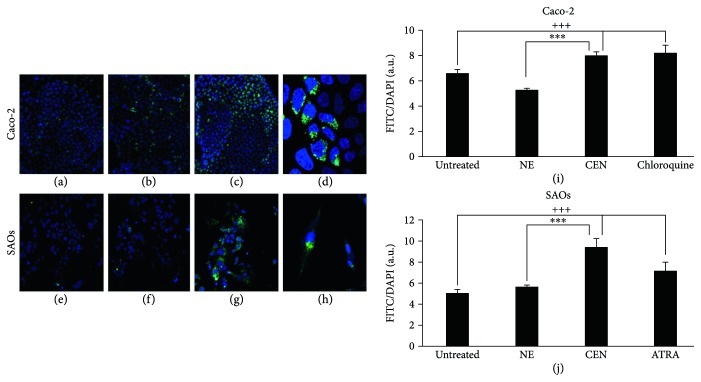
CEN induces autophagy in Caco-2 and SAOs cells. Representative images of autophagic vacuoles in Caco-2 and SAOs cells: untreated (a, e); after 168 h treatment with NE (b, f); or CEN (400 *μ*g/ml *w*/*v* for Caco-2 and 200 *μ*g/ml *w*/*v* for SAOs) (c, g), respectively. The photographs were taken with a confocal microscope (LEICA SP8) with a total magnification of 200x. A magnification at 400x of cells in (c, g) is reported in (d, h), respectively, to highlight the autophagic vacuoles (green) and cell nuclei (blue). Autophagosome quantification expressed as FITC/DAPI fluorescence ratio with Cyto-ID kit in Caco-2 (i) and SAOs (j) treated as described above using 20 *μ*M chloroquine and 2 *μ*M ATRA as positive controls, respectively. Bar graphs represent the mean ± SD; symbols indicate significance: ^∗∗∗^*p* < 0.001 with respect to NE and ^+++^*p* < 0.001 with respect to untreated cells.

Caco-2 and SAOs cells were treated with CEN, at a concentration of 400 and 200 *μ*g/ml (*w*/*v*), respectively, and compared to NE; subsequently, the autophagosomes were specifically stained with Cyto-ID green autophagy dye [24]. As positive control in Caco-2 cells, we employed chloroquine, a well-known reagent able to block autophagic flux and cause an accumulation of autophagosome in the cytoplasm [58], while in SAOs, we used ATRA, previously described as an autophagy modulator in this cell line [34].  Figure 4 indicates an increase in autophagosome formation after treatment with CEN, clearly visible with fluorescence microscopy (green fluorescence), in both Caco-2 (Figures 4(c) and 4(d)) and SAOs cell lines (Figures 4(g) and 4(h)), compared to untreated (Figures 4(a) and 4(e)) and NE treated cells (Figures 4(b) and 4(f)). The results of the CytoID autophagy assay were quantified as described in Materials and Methods. Figures 4(i) and 4(j) report the activation of autophagy by CEN, through a significant increase in autophagic vacuoles of about 1.5–1.7-fold, expressed as ratio FITC/DAPI, in Caco-2 (Figure 4(i)) and SAOs (Figure 4(j)), respectively, compared to untreated and NE treated cells.

**Figure 5:**
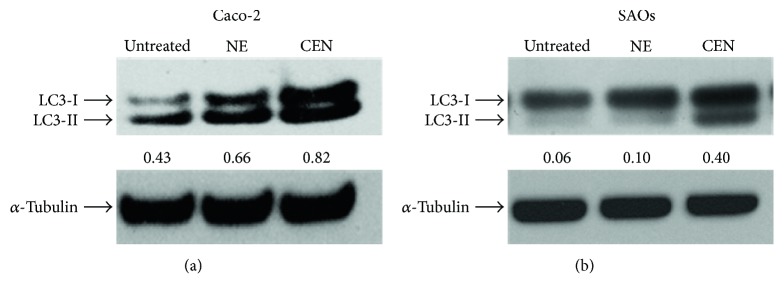
CEN increases expression of autophagic marker LC3-II. Western blot analysis of LC3-I/LC3-II expression in Caco-2 (a) and SAOs (b) cells treated for 168 h with NE and CEN (400 *μ*g/ml for Caco-2 and 200 *μ*g/ml for SAOs). Blots are representative of one out of three separate experiments performed. Densitometric analysis (numbers between panels) is expressed as the ratio between LC3-II and *α*-tubulin band intensities.

To further confirm the activation of autophagy induced by CEN, we evaluated the expression of the molecular marker LC3-II, the lipidated isoform of LC3 protein, essential in the autophagosome membrane formation [24]. The immunoblots and the corresponding densitometric analyses are reported in Figure 5, which shows the increased expression of LC3-II after 168 hours of incubation with CEN in Caco-2 (1.25-fold, Figure 5(a)) and in SAOs cells (4-fold, Figure 5(b)). Changes in LC3-II expression were also detectable in both cell lines at earlier times (96–120 h; data not shown).

### 3.3. The Carotenoid-Enriched Extract Activates a Nonprotective Form of Autophagy

Data obtained from Cyto-ID assays and LC3-II expression confirmed the involvement of autophagy in delaying cell proliferation after CEN treatment. Therefore, we further investigated this phenomenon trying to characterize the type of autophagy activated by CEN among those associated with cancer cell growth [27, 28]. We could easily exclude cytotoxic and cytostatic autophagy, characterized by induction of cell death and cell cycle arrest, respectively, since both processes were undetectable after CEN treatment (Figures 1 and 2). Similarly, we can *bona fide* rule out cytoprotective autophagy, since CEN did not promote cell survival and/or increase cell proliferation after several hours of treatment (Figures 1 and 2). Therefore, by exclusion, we hypothesized the activation of a nonprotective form of autophagy.

**Figure 6:**
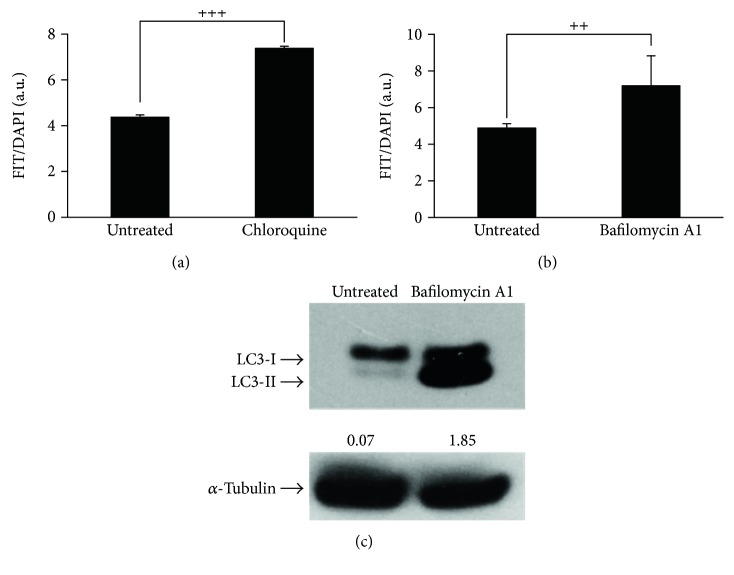
Chloroquine and bafilomycin A1 inhibit autophagy. Autophagosome quantification expressed as FITC/DAPI fluorescence ratio with Cyto-ID kit in SAOs treated with 20 *μ*M chloroquine (a) or 50 nM bafilomycin A1 (b) for 48 h. Bar graphs represent the mean ± SD with symbols indicating significance: ^++^*p* < 0.01 and ^+++^*p* < 0.001 with respect to untreated cells. (c) Immunoblotting showing LC3-I/LC3-II expression in SAOs cells treated with bafilomycin A1 as indicated above. Densitometric analysis values (numbers between panels) were expressed by the ratio LC3-II/*α*-tubulin.

To experimentally support this conclusion, we employed pharmacological inhibitors of autophagic flux [59]. To this regard, we firstly demonstrated that two canonical and broadly used autophagic inhibitors, namely, chloroquine and bafilomycin A1, were able to block the autophagic flux in SAOs cells. As reported in Figure 6, using Cyto-ID green autophagy dye, both compounds significantly inhibited autophagy demonstrated by the accumulation of autophagosomes in cells. In the case of the treatment with bafilomycin A1, we also detected an increased expression of LC3-II, the lipidated isoform of LC3 protein, as expected when a pharmacological inhibitor of autophagic flux is used, [24].

**Figure 7:**
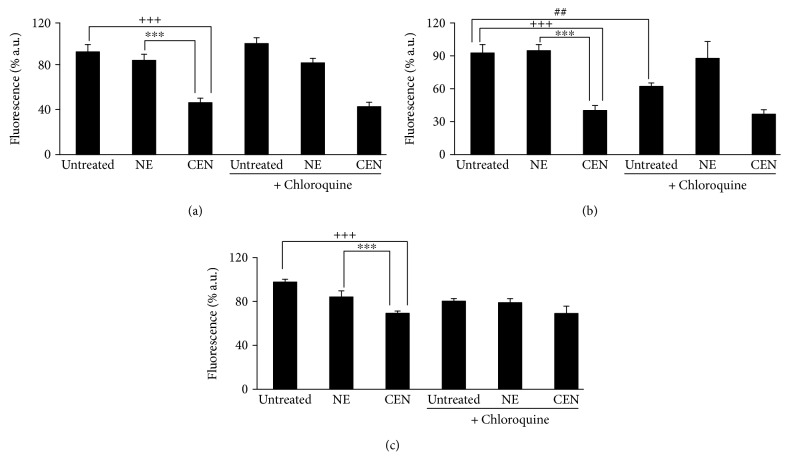
CEN induces nonprotective autophagy in SAOs (a, c) and Caco-2 (b) cells. Cells were treated for 144 h with different doses of NE and CEN (200 and 400 *μ*g/ml, *w*/*v*), respectively, following addition of 20 *μ*M chloroquine during the last 24 h (a), 48 h (b), or for 96 h following the addition of 50 nM bafilomycin A1 (c). CyQuant assay was performed at the end of incubation as described. Bar graphs represent the mean ± SD; symbols indicate significance: ^∗∗∗^*p* < 0.001 with respect to NE and ^+++^*p* < 0.001 with respect to untreated cells; ^##^*p* < 0.01 untreated versus untreated + chloroquine; no significant differences between CEN + chloroquine or CEN + bafilomycin A1 versus CEN alone.

To evaluate if the autophagy induced by CEN was protective or nonprotective, we designed the following experiment, based on the assumption that the inhibition of nonprotective autophagy does not affect drug sensitivity: in the presence of a nonprotective autophagy, after chloroquine inhibition, we should not detect any significant effect on cell growth comparing CEN + chloroquine versus CEN monotreatment. On the opposite, if “cytoprotective” autophagy was triggered by CEN, treating cells with CEN + chloroquine, the inhibition of the autophagic flux caused by chloroquine should result in an increased cytotoxicity (apoptosis). As shown in Figure 7(a), the cotreatment chloroquine + CEN failed to significantly modify the number of viable cells compared to CEN monotreatment. Therefore, we concluded that the activation of nonprotective autophagy in SAOs cells by CEN was responsible for the reduced rate of cell growth. Similar data with chloroquine were obtained in Caco-2 cells (Figure 7(b)). We repeated the same experiment using a different autophagic inhibitor, bafilomycin A1. As reported in Figure 7(c), also in this case, the cotreatment bafilomycin A1 plus CEN did not significantly modify the number of viable cells compared to CEN monotreatment, confirming the activation of a nonprotective form of autophagy in SAOs cells.

**Figure 8:**
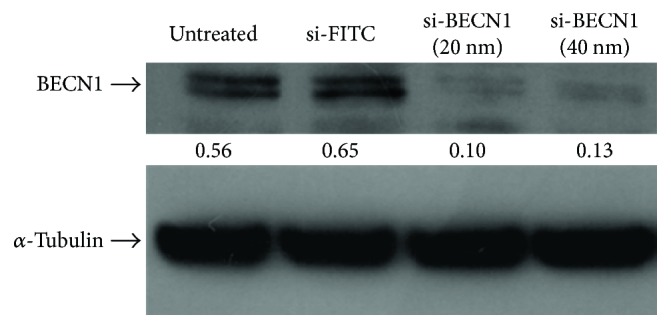
BECN1 silencing in SAOs cell line. Immunoblotting of BECN1 expression in SAOs cells untreated and treated for 72 h with si-FITC (40 nM) and si-RNA against BECN1 (20 nM and 40 nM). Blots are representative of one out of two separate experiments performed. Densitometric values reported between panels were expressed by the ratio BECN1/*α*-tubulin.

**Figure 9:**
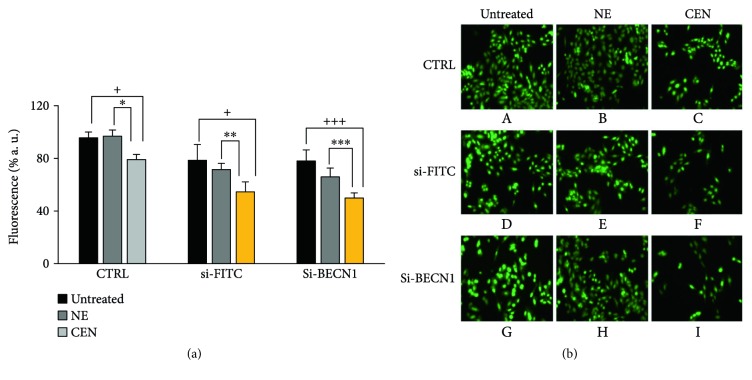
CEN induces nonprotective autophagy in SAOs cells. SAOs cells were treated with NE and CEN (200 *μ*g/ml, *w*/*v*) in the presence of a si-RNA against BECN1 (si-BECN1) or its corresponding control si-RNA (si-FITC, fluorescein conjugate) for 72 h. CyQuant assay was performed at the end of incubation as described in Materials and Methods. (a) Bar graphs represent the mean ± SD; symbols indicate significance: ^∗∗∗^*p* < 0.001, ^∗∗^*p* < 0.01, and ^∗^*p* < 0.05 versus NE and ^+++^*p* < 0.001 and ^+^*p* < 0.05 versus untreated cells; no significant differences were detected between si-BECN1 versus si-FITC. (b) Representative micrographs (microscope Axiovert 200 Zeiss; FITC fluorescence filter, 200x magnification) of cell nuclei stained with CyQuant fluorescent dye of SAOs untreated (A), (D), (G), after 72 h treatment with NE (B), (E), (H) and CEN (200 mg/ml, *w*/*v*) (C), (F), (I) (legend on top). The treatments with si-BECN1 and si-FITC described referred to (G), (H), (I) and (D), (E), (F), respectively (legend on the left).

To understand how CEN interferes with autophagic machinery, we used an approach different from the treatment with pharmacological autophagic inhibitors, that is, the genetic silencing of autophagy regulatory genes. To this aim, we selected BECN1/Beclin 1 (beclin 1, autophagy related), the mammalian homolog of yeast Vps30/Atg6 involved in the activation of macroautophagy [24]. As reported in Figure 8, we demonstrated that a si-RNA against BECN1 (si-BECN1) was able to almost abolish the expression of BECN1 dose dependently. When we repeated the treatment with CEN in SAOs cells in the presence of si-BECN1/si-FITC, we obtained a result superimposable to that described in Figure 7, where we used the pharmacological inhibitors. In fact, as reported in Figure 9, treating cells with CEN (Figure 9(a)), no significant differences in cell number comparing the si-BECN1 group of treatments versus the respective controls (si-FITC; compare the two orange bars in Figure 9(a)) were detected. These data were also confirmed by micrographs in Figure 9(b), where no significant differences in cell numbers are present comparing Figure 9(b), F with Figure 9(b), I.

### 3.4. CEN Blocks Autophagic Flux in SAOs

**Figure 10:**
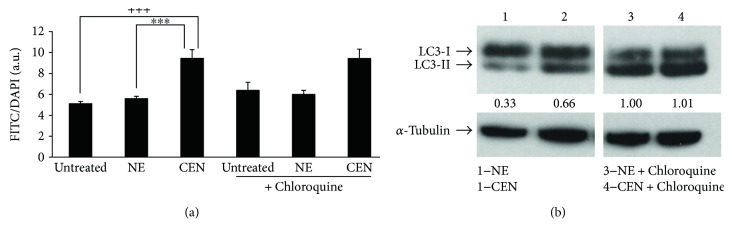
CEN inhibits SAOs autophagic flux. (a) Cells were treated with NE and CEN (200 *μ*g/ml, *w*/*v*) for 168 h with or without 20 *μ*M chloroquine added in the last 4 h of incubation. Autophagosome quantification was expressed as FITC/DAPI fluorescence ratio and obtained using Cyto-ID staining, as described. Bar graphs represent the mean ± SD; symbols indicate significance: ^∗∗∗^*p* < 0.001 with respect to NE and ^+++^*p* < 0.001 with respect to untreated cells; no significant differences between CEN + chloroquine versus CEN alone. (b) Immunoblotting of LC3-I/LC3-II in SAOs cells treated as in (a). Bands are representative of one out of three separate experiments performed. Densitometric analysis values (between panels) were expressed by the ratio LC3-II/*α*-tubulin.

To estimate the capacity of CEN to modulate the autophagic flux, we applied a well-described method consisting in adding the inhibitor chloroquine the last few hours before the end of the treatment of a supposed autophagic effector, that is, in our case, CEN and its respective control, NE. Subsequently, we measured autophagosome accumulation using Cyto-ID dye. As reported in Figure 10(a), the treatment did not induce any increase in the number of autophagosomes in the cells treated with CEN despite the presence/absence of chloroquine. If CEN would not block autophagic flux, we would expect an increase of Cyto-ID staining in the experiment corresponding to the treatment CEN + choloroquine. This behaviour was confirmed using LC3-II (Figure 10(b)). In fact, densitometric analyses show that the expression of LC3-II in lines 2 and 3 does not change significantly.

### 3.5. The Carotenoid-Enriched Extract Induces Cellular Differentiation

To understand how CEN could reduce the rate of cell growth and activate the nonprotective autophagy observed in Caco-2 and SAOs, we reasoned that both of these processes could be associated with the activation of cellular differentiation [60, 61]. We verified this hypothesis in SAOs cells, since they represented a suitable and reproducible cellular model to follow differentiation for the presence of well-characterized markers, such as changes in ALP activity and p27^kip1^ expression [62, 63].

**Figure 11:**
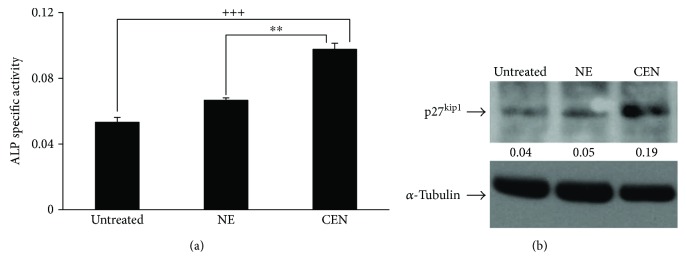
CEN induces differentiation in SAOs cells. ALP activity, used as differentiation marker, was assayed and expressed as specific activity in SAOs cells (a) after 120 h of treatment with NE and CEN (200 *μ*g/ml, *w*/*v*). ALP was determined as reported in Materials and Methods, and specific activity was expressed as O.D. values/min/*μ*g protein. Bar graphs represent the mean ± SD; symbols indicate significance: ^∗∗^*p* < 0.01 with respect to NE and ^+++^*p* < 0.001 with respect to untreated cells. Western blot analysis of p27^kip1^ protein expression in SAOs cells (b) treated as above. Densitometric analysis (numbers between panels) is expressed as the ratio between p27^kip1^ and *α*-tubulin band intensities.

Figure 11(a) shows that ALP enzymatic activity, measured after 120 h of treatment with CEN, was significantly higher (about 1.5-fold) with respect to untreated and NE-treated cells and lasted for the following 2 days (data not shown). Moreover, Figure 11(b) shows the increased expression of p27^kip1^ after 120 h of treatment with CEN (about 3-fold compared to NE-treated cells). These data confirm the hypothesis that autophagic induction and reduced cell proliferation could be functionally related to cellular differentiation. In addition, since osteosarcoma is a poorly differentiated tumour, the induction of differentiation using carotenoids or retinoids could represent an attractive therapeutic strategy, still little explored [64].

### 3.6. Biochemical Regulation of Autophagy by the Carotenoid-Enriched Extract

**Figure 12:**
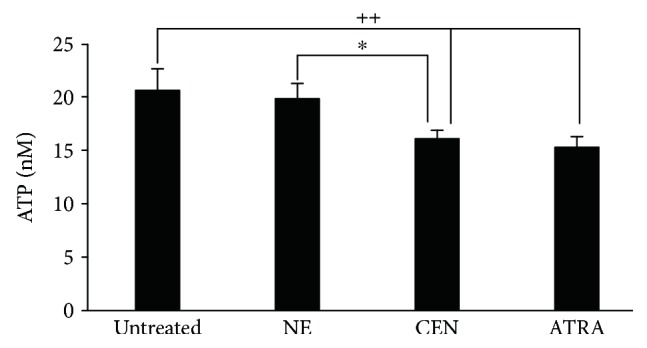
CEN modifies ATP intracellular levels in SAOs cells. Cells were treated for 24 h with NE, CEN (200 *μ*g/ml, *w*/*v*), and 2 *μ*M ATRA (positive control). ATP intracellular concentrations were measured as reported in Materials and Methods and expressed as nM ATP. Bar graphs represent the mean ± SD; symbols indicate significance ^∗^*p* < 0.05 with respect to NE and ^++^*p* < 0.01 with respect to untreated cells.

Energetic depletion is an important modulator of autophagy [23]. Therefore, we investigated whether nonprotective autophagy induced by CEN was related to an initial alteration of energetic metabolism. To this aim, intracellular ATP was measured as an index of functional integrity of metabolically active cells. As reported in Figure 12, the ATP intracellular levels, expressed as chemiluminescence value, significantly decreased after 24 hours of incubation with CEN in SAOs cells. It is worthwhile to note that the reduced ATP level was in the same range of the ATRA treatment, employed as positive control [65, 66].

**Figure 13:**
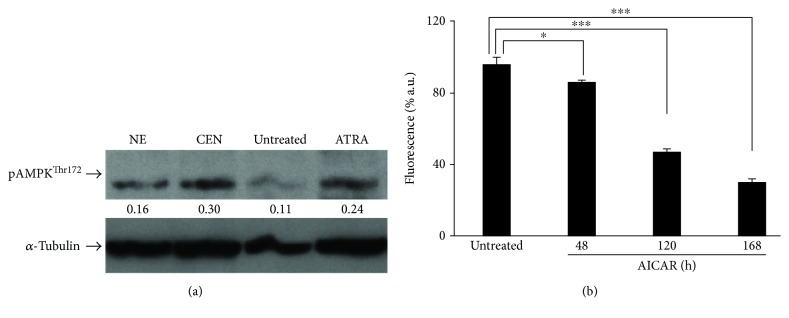
CEN reduces proliferation in SAOs cells upon AMPK activation. Western blot analysis of phosphorylated and active form (pAMPK^Thr172^) of AMPK in SAOs cells (a) treated for 24 h with NE and CEN (200 *μ*g/ml, *w*/*v*). ATRA (2 *μ*M) was used as positive control. Densitometric analysis (numbers between panels) is expressed as the ratio between pAMPK ^thr172^ and *α*-tubulin band intensities. AMPK activator, AICAR, reduces cell proliferation (b). Cells were incubated for the indicated times with 0.1 mM AICAR, and CyQuant assay was performed as described in Materials and Methods. Bar graphs represent the mean ± SD; symbols indicate significance ^∗^*p* < 0.05 and ^∗∗∗^*p* < 0.001 with respect to untreated cells.

Experimental evidence indicates that lowering ATP intracellular concentration leads to the activation of AMPK, a metabolic modulator of glucose and lipid metabolism which acts in response to the alterations of nutrients and ATP levels [45, 67]. In addition, AMPK is required for autophagic induction and is involved in the initial stage of autophagosome nucleation [68, 69]. For these reasons, we hypothesized that, in SAOs cells, autophagy could be triggered by a reduction of ATP induced by CEN, through the activation of AMPK. To this aim, we evaluated the expression levels of the phosphorylated and active form of the AMPK kinase, pAMPK^Thr172^. As reported in Figure 13(a), the phosphorylated protein increased twice after CEN treatment compared to controls. To further confirm the involvement of AMPK to explain CEN effects, we employed AICAR, a synthetic AMPK activator [45, 70].  Figure 13(b) shows that AICAR delayed cell growth time dependently in SAOs cells. This effect was already significant at 48 h, but became stronger at 120 and 168 h, mimicking CEN treatment.

The possibility that autophagy induced by CEN could be functionally related to cellular differentiation is supported by recent data reporting that AMPK is activated during osteoblast differentiation [60]. Moreover, it is well known that the energy sensing LK/AMPK pathway regulates p27^kip1^ phosphorylation and stabilization mediating the decision of cells to enter autophagy or apoptosis [71]. In particular, it has been suggested that early catabolic changes are important to provide the energy source for osteoblasts; furthermore, the activation of AMPK is a significant event for osteoblasts to progress until the final stage of differentiation [60].

An important novelty of the present work regards the potential impact of the nanoemulsion technology to improve solubility of the carotenoid-enriched extract, enhancing its stability and preventing degradation. Further studies are in progress to improve this technique.

**Figure 14:**
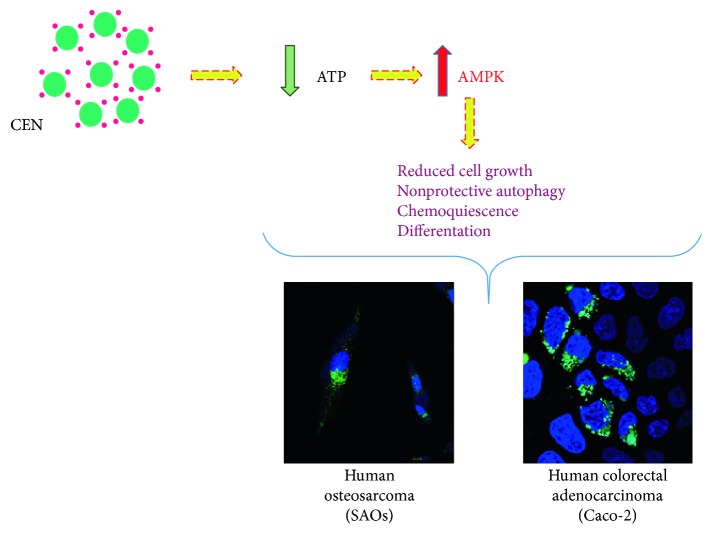
Cartoon summarizing the mechanism triggered by CEN to induce nonprotective autophagy via a decrease in ATP levels and activation of AMPK in the two cell lines investigated.

The results, summarized in Figure 14, show how CEN can delay cancer cell growth, leading to a cellular state recently defined “chemoquiescence” and associated with differentiation. This effect is always related to an alteration in energy metabolism and induction of nonprotective form of autophagy through the activation of AMPK or inhibition of mTOR/AKT-related signalling [72]. To our knowledge, data reported in this study represent one of the very few examples present in the literature where a carotenoid-derived preparation interferes with the autophagic process in malignant cell lines. Long time ago, in a short communication and in a totally different contest, before autophagy became known, it was described the capacity of carotenoids to slow the growth of small cell lung cancer cells [73].

This study suggests that a carotenoid-enriched extract from pumpkin *C. moschata* could be tested as therapeutic adjuvant in association with conventional drugs [58]. Data from previous studies on ATRA and vitamin D_3_ reinforce this hypothesis [34, 74]. In the next future, suitable animal model will be employed to test the chemopreventive and/or chemotherapeutic efficacy of the carotenoid-enriched nanoemulsions.

An interesting conclusion emerging from the present work is the observation that *β*-carotene from pumpkin, assayed as an individual compound, did not show any effects on cell proliferation at the concentration present in the nanoemulsions, suggesting, perhaps, a synergism among different components, or the presence of not yet identified bioactive molecules. Future work will shed light on this issue. Although the strategy to test vegetable extracts enriched with a specific class of biological compounds in preclinical cellular models is not novel and examples are abundantly present in the literature, we decided to apply this approach since the biological response of an extract may include potential additive/synergistic effects resulting in enhanced functional outcomes. This possibility reflects the observation that many bioactive molecules are present in the extract at relatively low concentrations, not enough to generate, per se, any measurable effect, but when associated with others can contribute to ameliorate a specific pathological or prepathological condition. The rationale of this hypothesis resides in the observation that, generally, biological extracts, as the one tested in the present work, contain compounds, which are structurally and functionally similar.

## 4. Conclusions

We investigated the potentiality of a carotenoid-enriched extract prepared from a local Italian cultivar of pumpkin to find applicative outcomes in the biomedical area using preclinical models. This possibility is supported by large body of scientific evidence that fruits and vegetables contain dietary chemopreventive agents, including vitamin A derivatives. We ended up with the observation that the carotenoid-containing preparation does not directly kill cancer cells, but delays their proliferation. In two different cell lines, Caco-2 and SAOs, the carotenoid-enriched extract, administered in the form of nanoemulsions, induced an alteration of the energetic metabolism which resulted in a decreased intracellular concentration of ATP and the activation of an autophagic process (Figure 14). We present evidence that the “nonprotective” form of autophagy, triggered by carotenoid-enriched nanoemulsions, directly or indirectly induces cellular differentiation in malignant cells, reducing their proliferating capacity.
